# CoDock-Ligand: combined template-based docking and CNN-based scoring in ligand binding prediction

**DOI:** 10.1186/s12859-023-05571-y

**Published:** 2023-11-23

**Authors:** Mingwei Pang, Wangqiu He, Xufeng Lu, Yuting She, Liangxu Xie, Ren Kong, Shan Chang

**Affiliations:** 1 Institute of Bioinformatics and Medical Engineering, School of Electrical and Information Engineering, Jiangsu University of Technology, Changzhou, 213001 Jiangsu China; 2 Primary Biotechnology Inc., Changzhou, 213125 Jiangsu China

**Keywords:** Ligand binding prediction, CASP, CoDock-Ligand, Molecular docking

## Abstract

For ligand binding prediction, it is crucial for molecular docking programs to integrate template-based modeling with a precise scoring function. Here, we proposed the CoDock-Ligand docking method that combines template-based modeling and the GNINA scoring function, a Convolutional Neural Network-based scoring function, for the ligand binding prediction in CASP15. Among the 21 targets, we obtained successful predictions in top 5 submissions for 14 targets and partially successful predictions for 4 targets. In particular, for the most complicated target, H1114, which contains 56 metal cofactors and small molecules, our docking method successfully predicted the binding of most ligands. Analysis of the failed systems showed that the predicted receptor protein presented conformational changes in the backbone and side chains of the binding site residues, which may cause large structural deviations in the ligand binding prediction. In summary, our hybrid docking scheme was efficiently adapted to the ligand binding prediction challenges in CASP15.

## Introduction

Ligand binding prediction is an important issue in the structure-based drug design. With the breakthrough of AlphaFold2 (AF2) in protein structure prediction [1], a new challenge category, ligand binding prediction was added to the 15th Community Wide Experiment on the Critical Assessment of Techniques for Protein Structure Prediction (CASP15). Previously, the Community Structure–Activity Resource (CSAR) [2] disseminated experimental datasets of diverse protein–ligand complexes to improve ligand docking and scoring. After 2015, the Drug Design Data Resource (D3R) [3] replaced CSAR and released valuable benchmarking datasets containing experimentally determined binding structures and affinity data. However, unlike the CSAR and D3R challenges, the ligand binding predictions of CASP15 provide only the target sequences without structural or ligand binding site information. These predictions are much more difficult and can be classified as blind docking. Unlike the typical blind docking, CASP only provides sequence information for the receptors. Therefore, it is necessary to predict the structures of these receptors. The predicted receptor structures may deviate from the experimental structures, regardless of the main chains or side chains, further increasing the difficulty of ligand blind docking. In addition, multiple ligands binding to one specific target make the problem even more complicated in CASP15.

Several computational methods have been developed for ligand binding prediction. Well-known ligand prediction programs include DOCK [4], AutoDock [5], Vina [6], Glide [7], GOLD [8], and MDock [9]. Recently, template-based methods have been widely used to predict ligand structures. Huang et al. proposed an enhanced Virtual Screening (VS) approach, EViS [10], which integrates ligand docking, protein pocket template searching, and ligand template shape similarity calculations. Zou et al. [11] proposed a new template-guided method using dissimilar ligands as templates, which significantly outperformed traditional molecular docking methods. PocketShape [12] used the Hungarian algorithm and the Downhill simplex method to solve the problem of binding site comparison, and achieved excellent performance in distinguishing similar from dissimilar ligand binding site pairs. To enrich the AlphaFold model with ligands and cofactors, AlphaFill [13] uses sequence and structural similarities to align small molecules and ions from experimentally determined structures with AF2 predicted protein models.

In addition, convolutional neural networks (CNNs) have been used in structure-based virtual screening and scoring. Ragoza et al. [14] showed that a fully CNN scoring function (GNINA scoring function) using only spatial and atom type information as input can outperform empirical and feature-based machine learning approaches for virtual screening. Deane et al. [15] used a Densely connected CNN (DenseNet) with a transfer learning approach to produce an ensemble of protein family-specific models for virtual screening. Jones et al. [16] fused models of 3D-CNNs and spatial graph neural networks (SG-CNNs) to make more accurate predictions than the previous docking scoring and MM/GBSA rescoring.

In CASP15, we participated in the category of ligand binding prediction. Owing to the advantages of template-based modeling and the GNINA scoring function, we combined these two methods to predict the binding modes of small molecules or metal ions. For most of the CASP15 ligand systems, our fusion docking protocol achieved successful or partially successful results. Considering its robust predictive performance, our docking protocol is a good alternative for the ligand binding predictions.

## Methods

### 3D alignment algorithm

In a previous work, we queried Protein Data Bank (PDB) [17] for template structures using sequence similarity searching. This sequence-based template search strategy has been used for protein–protein docking prediction [18, 19]. For ligand binding prediction, a structure-based 3D alignment algorithm was developed by our group and used for both pocket template searching and ligand alignment. For the pocket template searching, CA atoms in the protein pocket were set as nodes. The cutoff distance used to select CA atoms around the center of the ligand was 12 Å. For the ligand alignment, all atoms in the ligand except hydrogen were set as nodes. For the 3D alignment algorithm, a single graph representation was combined with the clique detection method. In a single graph representation, each node represents a pairing of atoms. One from the query structure and the other from the template structure. Adjacent nodes are two nodes for which both atoms from the query and template structures are separated by equivalent distances. First, a set of fully adjacent nodes is defined as a clique, which is a completely connected subgraph. Matching is then formulated as a graph theoretical problem that attempts to find completely connected subgraphs within an undirected graph. This 3D alignment algorithm is similar to the method used in the UCSF DOCK program [4]. In contrast to the exhaustive matching algorithm in the DOCK program, we used a greedy algorithm to improve speed, search for the most similar template, and generate orientation for alignment. We calculated the similarity coefficient as S_T_ = N_S_/N_T_, where N_S_ is the number of unique atoms shared between the query structure and the template structure, and N_T_ is the number of unique atoms in the template. The templates with S_T_ > 0.8 were defined as the high similar templates.

### GNINA scoring function

We used the GNINA scoring function to rescore the receptor-ligand complex [14, 20]. The GNINA scoring function is a CNN-based model [14]. The size of the box in this scoring function was 24 Å × 24 Å × 24 Å and was centered on the binding site with a default resolution of 0.5 Å. Each grid point stores information regarding the types of heavy atoms at that point. The ligand and protein atoms have distinct atom types, and each atom type is represented in a different channel (analogous to the RGB channels in the images) of the 3D grid. GNINA defined a total of 34 distinct types, with 16 receptor types and 18 ligand types. The new GNINA scoring function was trained using the PyTorch deep learning framework. The CNN model in the GNINA program was trained on the cross-docking and redocking datasets, and could predict both the pose score and binding affinity. The final ligand docking poses were evaluated and ranked using the GNINA scoring function.

### Docking protocol

**Fig. 1:**
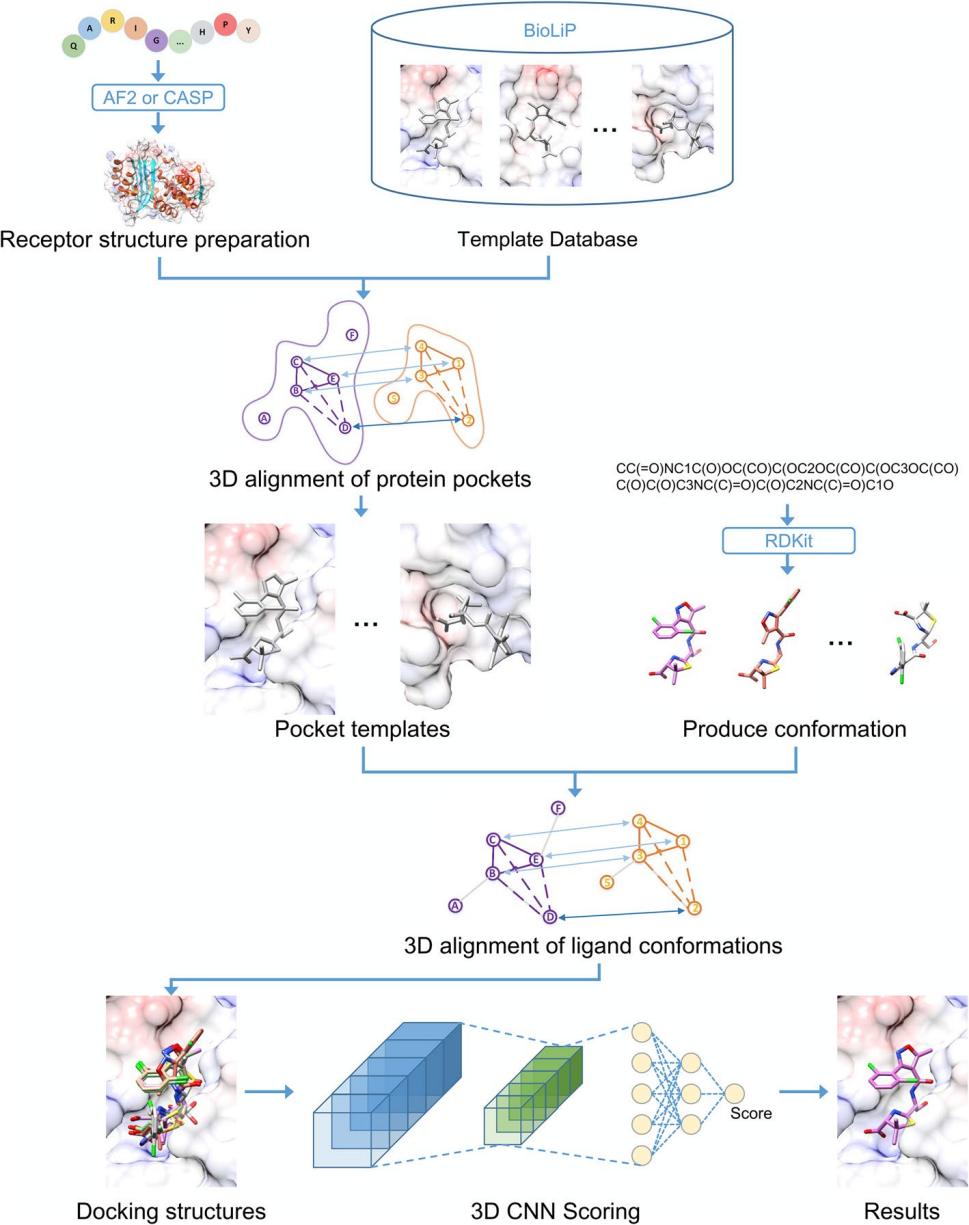
Flow chart of the CoDock-Ligand protocol. First, the receptor pocket templates are searched in the template library using the pocket 3D alignment algorithm. Second, multiple ligand conformations are generated using RDKit. Finally, the ligand conformations are aligned to the small molecules from the pocket template, and the variety binding poses are sorted by the GNINA scoring function

Template-based modeling and the GNINA scoring function were combined for ligand binding prediction, and the flow chart is shown in Fig. 1. For each target, the receptor structure was predicted using AF2, or that disclosed by the CASP Organizing Committee. We queried BioLiP [21] using the 3D alignment algorithm and extracted potential pocket templates for the receptor. BioLiP is a widely used databases for protein–ligand interactions, and the data were primarily collected from the Protein Data Bank (PDB). Because ligand molecules are often used as additives to solve protein structures, not all ligands present in the PDB database are biologically relevant. BioLiP uses a composite of automated and manual procedures to examine the biological relevance of ligands. Therefore, BioLiP is very useful for template-based protein–ligand docking. If an appropriate template was available, the initial ligand binding pocket was identified based on the template. The ligand conformations were then generated from the SMILES string using RDKit [22]. The ligand conformations were aligned to small molecules of the template using the 3D alignment algorithm to generate various docking poses. Finally, these binding poses were evaluated using the GNINA scoring function. Therefore, the box size in the docking protocol was the same as that of the GNINA scoring function. We provided a web server to users to facilitate the use of our docking program (https://codockligand.schanglab.org.cn).

## Results and discussion

### Docking protocol test

**Fig. 2:**
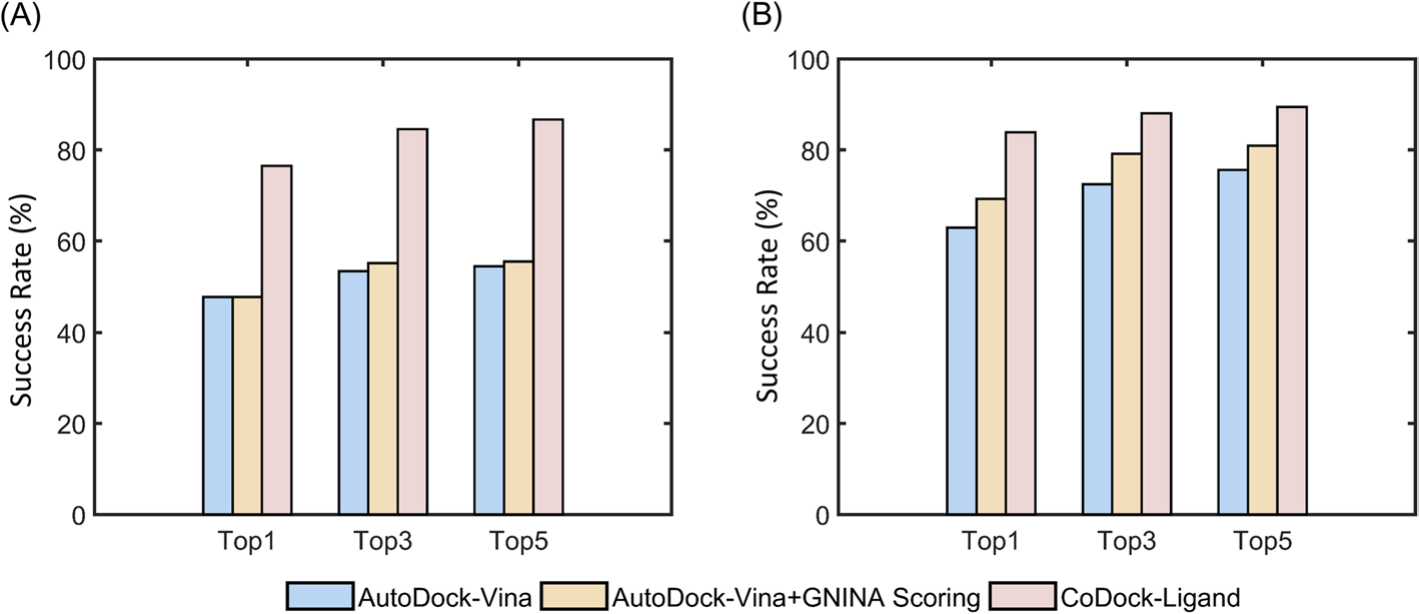
Histogram of the comparison between AutoDock-Vina, AutoDock-Vina + GNINA scoring and CoDock-Ligand in the CASF-2016 dataset. **A** Success rates of three programs with RMSD ≤ 1.0 Å. **B** Success rates of three programs with RMSD ≤ 2.0 Å

Upon participating in the ligand prediction assessment of CASP15, we standardized the algorithm as a docking protocol. It was tested on CASF-2016 [23], and compared with the widely used AutoDock-Vina program [24]. We also compared the test results of the combination of AutoDock-Vina and GNINA scoring (AutoDock-Vina + GNINA scoring). The docking box of AutoDock Vina was defined as the center of the native ligand coordinates with dimensions of 28 Å × 28 Å × 28 Å to include the residues of the entire cavity. The exhaustiveness value was 10. The root-mean-square deviation (RMSD) was calculated for all non-hydrogen atoms in the ligand relative to the native structure. To avoid introducing biases into the docking tests, we removed the overlapping systems between BioLiP and the test set of CASF-2016. As shown in Fig. 2, CoDock-Ligand achieved better performance than AutoDock-Vina and AutoDock-Vina + GNINA scoring in terms of the success rates of the top ranking poses. For the Top1 pose, 76.5% of the systems were predicted successfully using CoDock-Ligand with RMSD ≤ 1 Å, and 83.9% of the systems were predicted successfully with RMSD ≤ 2 Å. Correspondingly, the success rates of AutoDock-Vina were 47.7% and 62.9%, respectively. Although the GNINA scoring function was the same, the success rates of AutoDock-Vina + GNINA scoring were 47.7% and 69.3%, respectively. These comparisons demonstrated the advantages of incorporating experimental data during docking.

### The overview in CASP15

**Fig. 3:**
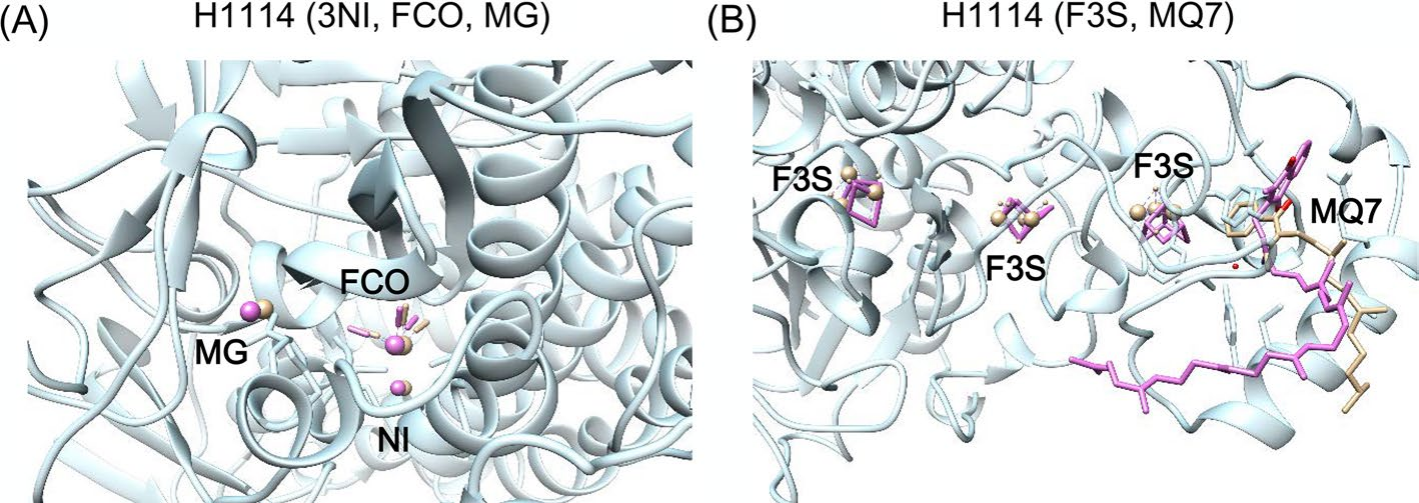
Ligand prediction of H1114. The receptor protein and ligands of the crystal structure are colored light blue and orange, respectively. The predicted ligand structures are colored pink. **A** Ligands of Ni ion, FCO, and Mg ion. **B** Ligands of F3S and MQ7

**Fig. 4:**
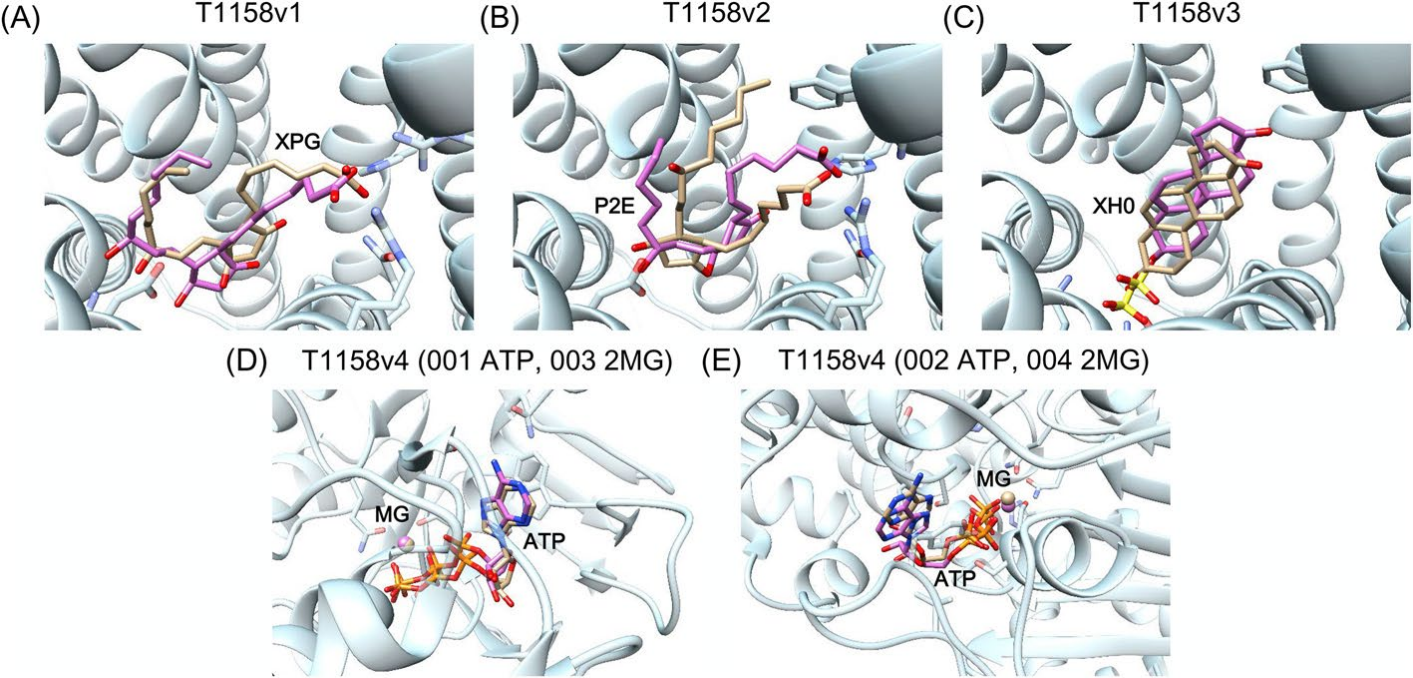
Prediction of four different ligand binding with T1158. The receptor protein and ligands of the crystal structure are colored light blue and orange, respectively. The predicted ligand structures are colored pink. **A** Ligand of T1158v1. **B** Ligand of T1158v2. **C** Ligand of T1158v3. **D** Ligands of 001 ATP and 003 Mg ion in T1158v4. **E** Ligands of 002 ATP and 004 Mg ion in T1158v4

**Fig. 5:**
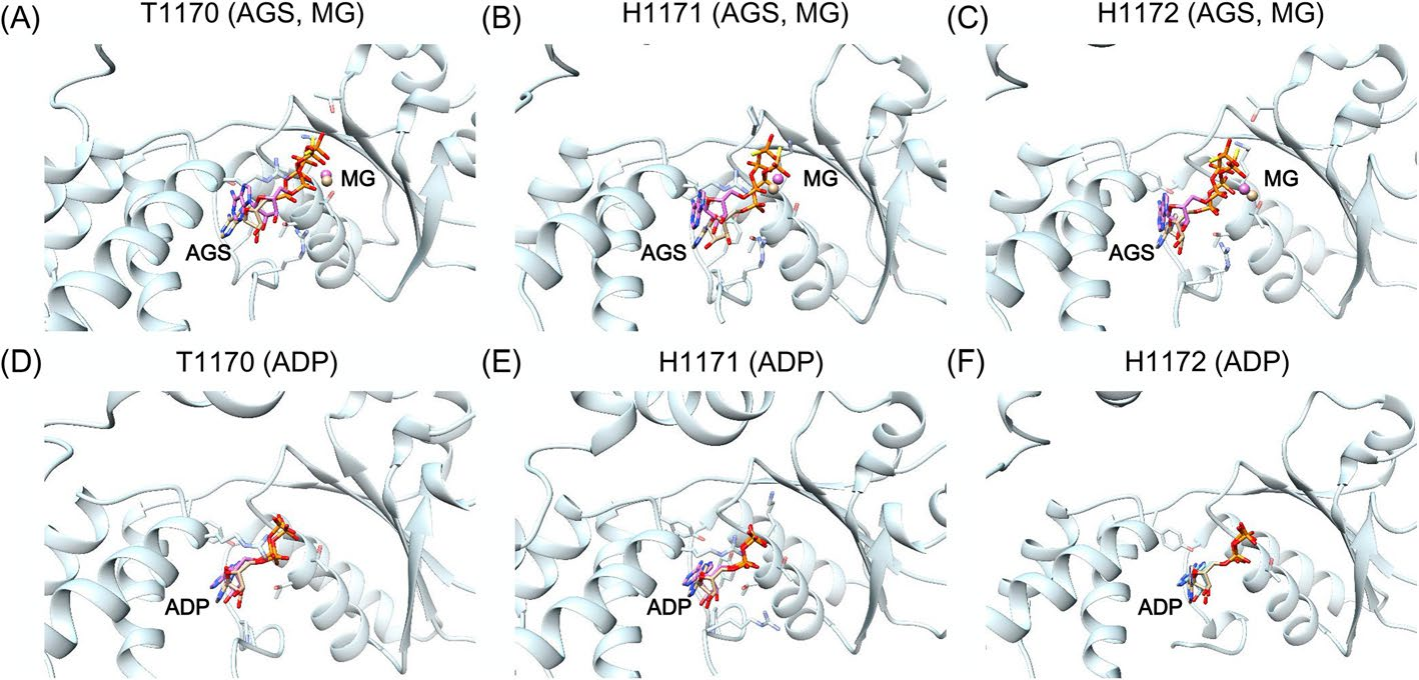
Ligand prediction of T1170-H1172. The receptor protein and ligands of the crystal structure are colored light blue and orange, respectively. The predicted ligand structures are colored pink. **A**, **D** are ligands of T1170. **B**, **E** are ligands of H1171. **C**, **F** are ligands of H1172

**Fig. 6:**
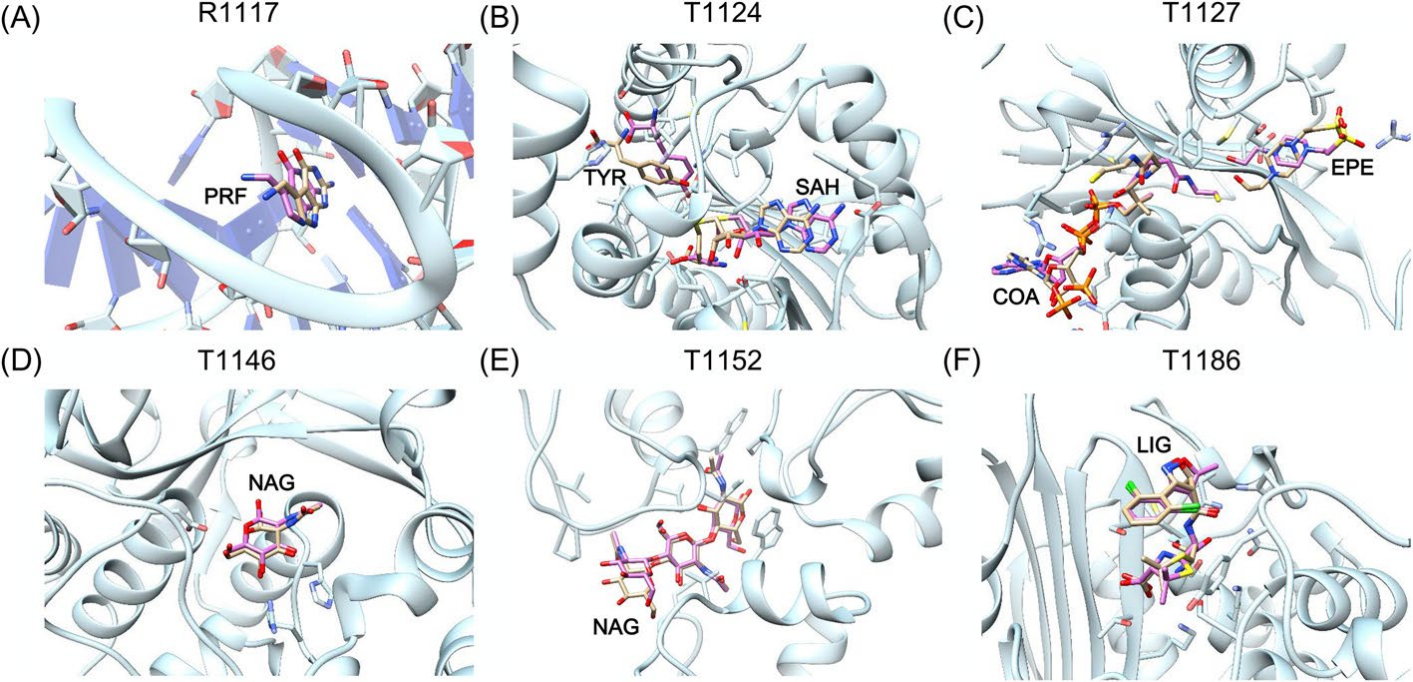
Successful ligand prediction of six systems. The receptor protein and ligands of crystal structure are colored light blue and orange, respectively. The predicted ligand structures are colored pink. **A** Ligand of R1117. **B** Ligands of T1124. **C** Ligands of T1127. **D** Ligand of T1146. **E** Ligand of T1152. **F** Ligand of T1186

**Fig. 7:**
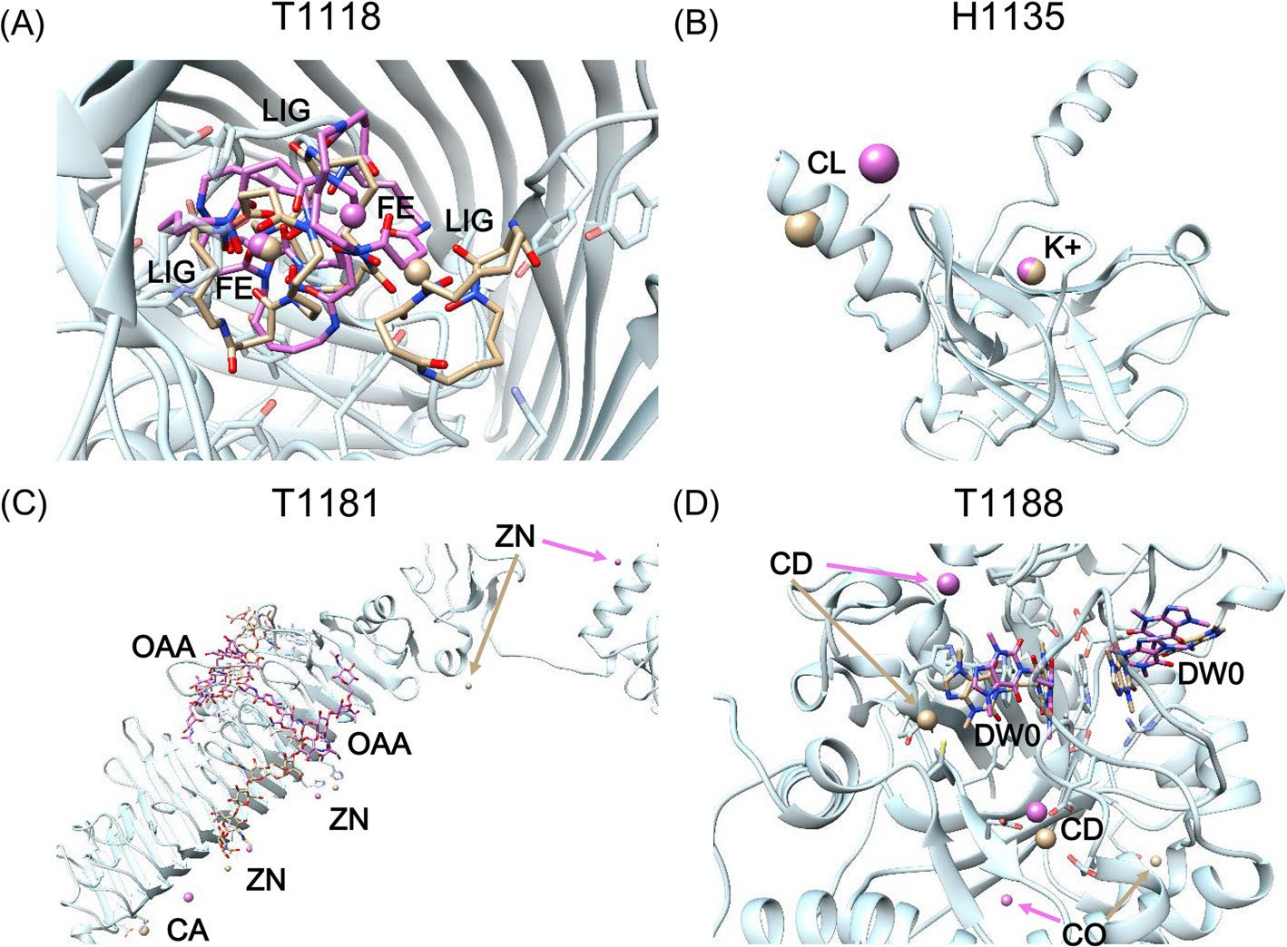
Partial successfully ligand prediction of four systems. The receptor protein and ligands of crystal structure are colored light blue and orange, respectively. The predicted ligand structures are colored pink. **A** Ligands of T1118. **B** Ligands of H1135. **C** Ligands of T1181. **D** Ligands of T1188

**Fig. 8:**
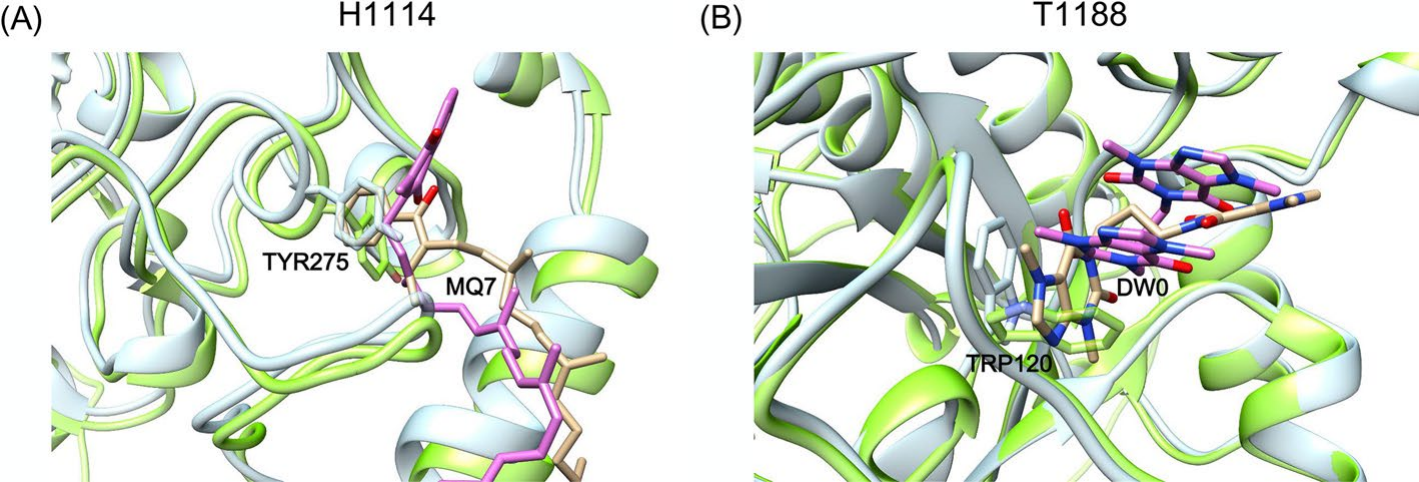
Analysis the effect of the side chain on ligand prediction. The crystal structures of receptor protein and ligand are colored light blue and orange, respectively. The predicted structures of receptor protein and ligand are colored light green and pink, respectively. **A** Prediction of MQ7 in H1114. The side chain of TYR275 leads to the incorrect conformation of MQ7. **B** Prediction of DW0 in T1188. The side chain of TRP120 leads to the orientation change of the aromatic ring in DW0

**Fig. 9:**
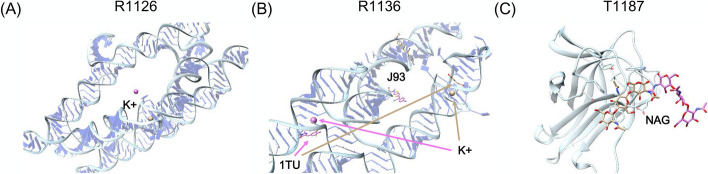
Failed ligand prediction of three systems. The receptor protein and ligands of crystal structure are colored light blue and orange, respectively. The predicted ligand structures are colored pink. **A** Ligands of R1126. **B** Ligands of R1136. **C** Ligands of T1187

Considering that many targets in CASP15 are multimers with duplicate subunits, we only take one subunit as an example to show ligand binding prediction. For each target, five models were submitted with five different poses in each model. There were 25 binding poses for each ligand, and RMSD values were calculated to reference the experimental structures. The lowest RMSD values of the 25 binding poses for each target are listed in Table 1. These results are consistent with those shown in Figs. 3, 4, 5, 6, 7, 8 and 9, and are convenient for comparing the predicted ligands with their crystal structures. In Table 1, high quality predictions are defined as RMSD ≤ 2.0 Å, accepted site predictions as 2.0 Å < RMSD ≤ 5 Å and failed predictions as RMSD > 5.0 Å. The CASP only provides sequence information for the receptors. The predicted receptor structures may deviate significantly from the experimental structures, regardless of the main chains or side chains, further increasing the difficulty of ligand docking. The prediction with RMSD between 2 and 5 Å is also important, showing that the site is correct but pose is not. In previous studies [11, 25], the RMSD cutoff of 5 Å was used as the criterion.

**Table 1:** Performance of CoDock-Ligand in CASP15

CASP ID	Template	Oligomeric state	Ligand	Lowest RMSD		
				L_RMSD ≤ 2.0 Å	2.0 Å < L_RMSD ≤ 5.0 Å	L_RMSD > 5.0 Å
H1114^a^	5Y4N, 4UE3, 4KO2, 2FRV	A4B8C8	7/56	0.65(001/3NI) 1.20(009/F3S) 0.56(017/F3S) 0.69(025/F3S) 0.82(033/FCO) 1.28(041/MG)	3.87(049/MQ7)	
R1117	2L1V, 3FU2	A1	1/1	1.17(001/PRF)		
*T1118*^b^	5JJ5,6Z8A	A1	5/9	0.40(002/FE)	4.15(001/FE) 4.35(004/LIG) 4.73(005/LIG)	9.13(003/LIG)
T1124	7WDW	A2	2/4	1.92(001/SAH)	2.71(003/TYR)	
R1126	5OB3, 7L0Z	A1	1/1			27.29(001/K)
T1127	3BJ7, 3BJ8, 2B4D, 2B4B, 2JEV, 2B58	A2	2/5		3.17(001/COA) 2.16(003/EPE)	
*H1135*	6R16, 6R15, 6R2I	A9B3	2/12	0.08(004/K)		11.32(001/CL)
R1136	4KZD, 5OB3	A1	3/3			72.05(001/1TU) 24.27(002/J93) 64.57(003/K)
T1146	4Q5K, 4Q68	A1	1/1	0.58(001/NAG)		
T1152	4B8V	A2	1/1	0.81(001/NAG)		
T1158v1	5UJA, 6D3R, 6PZ9, 6PZI, 6UY0	A1	1/1	1.85(001/XPG)		
T1158v2	5UJA, 6D3R, 6PZ9, 6PZI, 6UY0	A1	1/1		3.90(001/P2E)	
T1158v3	5UJA, 6D3R, 6PZ9, 6PZI, 6UY0	A1	1/1	1.66(001/XH0)		
T1158v4	5UJA, 6D3R, 6PZ9, 6PZI, 6UY0	A1	4/4	0.79(001/ATP) 1.24(002/ATP) 0.14(003/2MG) 0.65(004/2MG)		
T1170	6CHS	A6	3/9	1.20(001/ADP) 0.77(007/MG)	2.45(004/AGS)	
H1171	6CHS	A6B1	3/9	1.52(001/ADP) 1.57(007/MG)	2.48(004/AGS)	
H1172	6CHS	A6B2	3/9	1.06(007/MG)	2.09(001/ADP) 2.38(004/AGS)	
*T1181*	5W6H, 4OJ6, 4OJ5, 4OJP, 4OJO	A3	6/8		4.45(006/ZN)	10.63(001/OAA) 16.33(002/OAA) 36.99(005/ZN) 5.69(007/ZN) 27.23(008/CA)
T1186	1FCM, 4R1G	A1	1/1	1.05(001/LIG)		
T1187	–	A2	1/2			14.36(001/NAG)
*T1188*	2YBT, 6BT9	A1	5/5		3.96(001/DW0) 2.05(004/CD)	6.02(002/DW0) 11.01(003/CD) 15.42(005/CO)

^a^In these targets, our group achieved successful results in ligand binding predictions

^b^In these targets, our group achieved partially successful results in ligand binding predictions

For some target systems, complex templates with high similarity scores were identified, including H1114, R1117, T1124, T1127, H1135, T1146, T1152, T1158, T1170, H1171, H1172 and T1186. For ligands identical to those in the complex template, structure-based alignment was directly used to obtain the ligand position in the predicted target. For example, the ligand of R1117 and metal ions of H1114 were predicted in this manner. For the ion ligands, a simple coordinate transformation was used for docking prediction. For ligands chemically similar to those in the complex templates, template guided docking protocol was used to obtain the target-ligand complex structure. It was applied for the ligand predictions of T1124, T1158 v1, T1158 v2, and T1152. For target systems without appropriate complex templates, such as T1181 and T1187, traditional docking was performed using Glide [7]. Previous study compared Glide and GNINA on the CASF-2016 dataset [25], and demonstrated that Glide performed slightly better than GNINA. When no acceptable template structures were found, Glide was used for docking.

### Successfully predicted targets

#### H1114

H1114 is the [NiFe]-hydrogenase Huc from *Mycobacterium smegmatis* (PDB: 7UUS) [26]. The Huc catalytic subunits form an octameric complex containing 56 metal cofactors and small molecules. For this system, 5Y4N, 4UE3, 4KO2, and 2FRV were selected as the templates. Six metal cofactors and one small-molecule compound, MQ7, were predicted for each subunit. As shown in Fig. 3, the binding poses of six metal cofactors were predicted correctly with RMSD < 2.0 Å. For the MQ7 ligand, the binding site was correct, but the lowest RMSD was 3.87 Å.

#### T1158

T1158 v1 to v4 are MRP4-mut E1202Q protein in four states, binding to four different ligands, respectively. It is a multidrug resistance-associated protein that transport compounds out of cells. 5UJA, 6D3R, 6PZ9, 6PZI, and 6UY0 were identified as highly similar templates and used for ligand binding prediction. Based on the protein function and ligand binding in the template structures, it was deduced that the binding site was located in the center channel of the protein. As shown in Fig. 4, the ligands in v1, v3, and v4 states were predicted correctly with RMSD < 2.0 Å. For the P2E ligand in v2 state, the binding site was predicted correctly, but the lowest RMSD was 3.9 Å.

#### T1170-H1172

T1170-H1172 are RuvAB branch migration motor in complex with the Holliday junction (PDBs: 7PBR, 7PBL, and 7PBP) [27]. These systems bind to the same ligands, including AGS, ADP, and Mg ion. The only difference is that H1171 and H1172 are in different multimer states. To simplify the analysis, we compared only the ligands in one of these states. For these systems, 6CHS was selected as the template. As shown in Fig. 5, AGS, ADP, and Mg ion were correctly predicted in these three systems. Most of ADP and Mg ion were predicted correctly with RMSD < 2 Å. However, AGS has more rotatable bonds than ADP, and its lowest RMSDs in these three systems were 2.45, 2.48 and 2.38 Å, respectively.

#### Other six systems

The four systems, R1117, T1146, T1152, and T1186, are relatively simple, with only one ligand to be predicted. R1117 is a PreQ1 class I type III riboswitch, and we selected 2L1V and 3FU2 as the templates. For the T1146 system, 4Q5K and 4Q68 were used as the templates. T1152 is a clostridium thermocellum CtCBM50 structure in complex with beta-1,4-GlcNAc trisaccharide (PDB: 7R1L), and we used 4B8V as the template. T1186 is a beta-lactamase with dicloxacillin, and we selected 1FCM and 4R1G as the templates. As shown Fig. 6A, D–F, the ligand structures of these four systems were correctly predicted, and the lowest RMSDs were 1.17, 0.58, 1.52 and 1.05 Å, respectively.

The other two systems, T1124 and T1127, were more complex than the above four systems, and both had two ligands to be predicted. T1124 is an L- and D-tyrosine O-methyltransferase from the marformycin biosynthesis pathway of Streptomyces drozdowiczii, with SAH and L-tyrosine bound (PDB: 7UX8) [28]. For this system, 7WDW was used as the template. For the T1127 system, 3BJ7, 3BJ8, 2B4D, 2B4B, 2JEV, and 2B58 were selected as the templates. As shown Fig. 6B, C, the lowest RMSDs of the ligands in these two systems were close to 2.0–3.0 Å.

### Partial successfully predicted targets

#### Four systems

T1118 is an outer membrane FoxA with (2:3) Fe-bisucaberin bound. According to the CASP information, the FoxA structure in complex with another ligand, nocardamine, has been solved (PDB: 6Z8A) [29]. For this system, 5JJ5 and 6Z8A were used as the templates. As shown in Fig. 7A, the binding sites of the ligand were correctly predicted, but the orientations of the two ligands (a Fe ion and a bisucaberin) were far away from the crystal structure. The RMSDs were 4.15 and 9.13 Å, respectively.

The oligomeric state of H1135 was A9B3, and we took one subunit for analysis (see Fig. 7B). For this system, 6R16, 6R15, and 6R2I were used as the templates. The K ion was functionally relevant and its predicted deviation was less than 1.0 Å. Although the pose of Cl ion was incorrect, it was not functionally relevant.

T1181 is a trimer, and one subunit was shown for the ligand binding prediction in Fig. 7C. For this system, 5W6H, 4OJ6, 4OJ5, 4OJP, and 4OJO were used as the templates. The ligand OAA is a polysaccharide molecule containing 47 rotatable bonds. Thus, only one Zn ion were predicted with RMSD < 5.0 Å, but the ligands OAA and Ca ion were incorrectly predicted.

T1188 had five ligands to be predicted, including two DW0 ligands and three metal cofactors. For this system, 2YBT and 6BT9 were used as the templates. As shown in Fig. 7D, the poses of two DW0 and a Cd ion in T1188 were basically correct, and the RMSDs were 3.96, 6.02 and 2.05 Å, respectively. However, the lowest RMSDs of the other two metal ions, Cd and Co ions were 11.01 and 15.42 Å, respectively.

In the above successful and partial successfully prediction targets, we also determined the reasons why some ligands were located in the correct binding pocket but had a large RMSD value. Because the receptor structure was predicted, the side chain orientations or main chain conformations of the receptor were different between the predicted structure and the experimental structure. In some cases of CASP15, subtle rotamer rearrangements of side chains greatly affect the docking results and lead to incorrect predictions of ligand poses, especially for those with π–π interactions. As shown in Fig. 8, the side chain orientations of TYR275 and TRP120 led to remarkable deviations in the ligand predictions for MQ7 in H1114 and DW0 in T1188. For the T1188 system, the RMSD value was still larger than 5.0 Å despite the correct predicted site of DW0.

### Failed prediction targets

#### Three systems

The target structures of R1126 and R1136 are RNA molecules in the Traptamer and Apta-FRET forms, respectively. The ligand of R1126 is K ion, and those of R1136 are 1TU, J93, and K ion, respectively. We found some templates with similar ligands for these two systems. However, we used the receptor structures provided by the CASP for RNA targets. Actually, the final evaluation shows that the backbone deviations between these predicted receptor structures and the experimental structures were 52.637 and 54.508 Å, respectively. Thus, the ligands could not be aligned correctly by our modeling method (see Fig. 9A, B).

Another failed target is T1187, which is a tobacco lectin Nictaba in complex with triacetylchitotriose (PDB: 8AD2). For this system, our docking protocol did not identify any appropriate complex templates, so traditional docking was performed using Glide. As shown in Fig. 9C, the predicted binding site deviated from the actual binding site, and the lowest RMSD was 14.36 Å.

Here we proposed a template based docking protocol, CoDock-Ligand, and applied in CASP15 Ligand prediction category. An atom-type based 3D align algorithm was designed to capture potential pocket templates and perform ligand alignment. Combined with GNINA scoring function, CoDock-Ligand achieved better performance than AutoDock-Vina in terms of the success rates of ligand poses. Our group (CoDock) was ranked as No. 1 in the ligand prediction category, showing remarkable accuracy in receptor-ligand complex structure prediction. However, the receptor structure showed significant impact for the ligand binding predictions. Such as the ligand predictions for MQ7 in H1114 and DW0 in T1188, subtle side-chain conformation changes of pocket residues made the bad predictions for ligand poses. For RNA-ligand interactions, poor RNA structure predictions led to final failures for ligand binding predictions. It is necessary to consider the conformation flexibility of proteins and further understand of RNA interactions [30–34], which may help to improve the ligand binding predictions in the future.

In recent years, ODE-based theoretical modeling studies have been widely applied on gene/protein signaling networks [35–37]. Combine with these methods, the docking models could contribute to understand regulatory mechanisms and find potential therapeutic targets in diseases. In addition, some new computational methods, such as graph convolutional neural networks, have been used to predict interactions, which provide valuable insights into genetic markers and related diseases [34, 38, 39]. These models will be helpful to improve the docking protocol and further provide atomic details for interactions between bio-molecules.

## Conclusion

Our docking protocol combines template-based modeling and the GNINA scoring function for receptor-ligand structure prediction. The template-based modeling method adopts a structure-based 3D alignment algorithm developed by our group that can accurately identify templates from the structure database. This method captured similar templates for most targets, and CoDock-Ligand achieved successful or partially successful predictions for these systems in CASP15. Meanwhile, we analyzed the failed systems to determine the reasons. If there was a remarkable backbone deviation between the predicted receptor structure and the experimental structure, such as the RNA-ligand systems R1126 and R1136, our docking protocol failed in the ligand binding prediction. Because the receptor structures are predicted in the ligand prediction assessment of CASP15, the side chain conformations, especially the orientations of the aromatic rings have a significant impact on ligand binding predictions. Therefore, the flexibility of the receptor should be considered in the docking protocol, and the precise receptor structure greatly contributes to ligand binding predictions.

## Data Availability

The data of CASP15 experiment can be downloaded from http://predictioncenter.org/casp15/index.cgi. The CoDock-Ligand web server, with the online documentation, is publicly available at https://codockligand.schanglab.org.cn.

## Abbreviations

GNINA: A fork of SMINA that allows the utilization of grid-based CNN models as scoring functions
CASP15: The 15th Community Wide Experiment on the Critical Assessment of Techniques for Protein Structure Prediction
CNN: Convolutional neural network
CSAR: The Community Structure–Activity Resource
D3R: Drug Design Data Resource
VS: Virtual Screening
DenseNet: Densely connected CNN
SG-CNNs: Spatial graph neural networks
PDB: Protein Data Bank
